# Clinical Study on Treatment of Acute Lower Extremity Arterial Embolism With Straub Thrombus Removal System

**DOI:** 10.3389/fsurg.2022.891649

**Published:** 2022-04-29

**Authors:** Liang Zhao, Hui Cai, Qiang Song

**Affiliations:** ^1^Department of Vascular Surgery, The First Affiliated Hospital of Xi 'an Jiaotong University, Xi 'an, China; ^2^Department of Structural Heart Disease, The First Affiliated Hospital of Xi 'an Jiaotong University, Xi 'an, China

**Keywords:** acute lower extremity arterial embolism, straub thrombus removal system, minimally invasive interventions, influencing factor, curative effect

## Abstract

**Background:**

Acute lower extremity arterial embolism (ALEAE) is a common and frequently occurring disease in clinics. Although thrombectomy with arteriotomy has been widely used and developed in clinics, there is a high probability of embolic recurrence after operation. The present study investigated the clinical efficacy of the Straub Rotarex system in the treatment of ALEAE, as it could remove exfoliative substances in acute and chronic cavities and expose diseased vessels.

**Materials and Methods:**

We accessed our institutional database and retrospectively screened all patients with ALEAEs who received surgical treatment between April 2018 and April 2021. To observe the clinical efficacy, surgical indicators, incidence of postoperative complications, and recurrence rate of treatment with Straub Rotarex system and arteriotomy thrombectomy and analyze the risk factors for recurrence of embolism after treatment with Straub Rotarex system by multivariate Logistic regression model.

**Results:**

Finally, 64 patients were included as the research object. The total effective rates of the observation group and the control group after operation were 100 and 93.75% respectively, and there was no significant difference between the two groups (*P* > 0.05). The intraoperative blood loss, postoperative off-bed time and hospital stay time in the observation group were significantly lower than those in the control group, and the operation time and hospitalization expenses were significantly higher than those in the control group (*P* < 0.05). The incidence of postoperative complications in the observation group was 3.13%, which was significantly lower than 18.76% of that in the control group (*P* < 0.05). The recurrence rates of the observation group and the control group were 15.63 and 18.76%, respectively. There was no significant difference in the recurrence rate between the two groups *(P* > 0.05). Atrial fibrillation was an independent risk factor for recurrence after the Straub thrombus removal system (*P* < 0.05).

**Conclusion:**

Straub thrombus removal system is an effective method for the treatment of ALEAE. Although it prolongs the operation time and increases the operation cost as compared with thrombectomy, it effectively improves the operation safety, postoperative life quality, and postoperative recovery, thus, worthy of clinical promotion. Atrial fibrillation is an independent risk factor for recurrent embolism after the Straub thrombus removal system. Paying attention to the clinical diagnosis and treatment of patients with atrial fibrillation is of great significance for patients to choose a reasonable treatment, prevent a recurrence, and improve the prognosis.

## Introduction

Acute lower extremity arterial embolism (ALEAE) is a vascular surgical emergency caused by thrombus, atherosclerotic plaque, and other emboli falling off the heart or artery wall flowing with the blood and forming embolism at the artery of the lower extremity (1). There are many causes for the formation of AE, most of which are caused by slow blood flow and changes in blood viscosity of such patients, based on vascular stenosis caused by atherosclerotic plaques. Arteriotomy and thrombectomy is a common method for the treatment of ALEAE, which can effectively reduce the disability rate and mortality rate of patients with ALEAE. However, it also has the disadvantages of large trauma of thrombectomy and large postoperative reperfusion reaction (2). The Straub Rotarex system is suitable for the treatment of acute and chronic arterial thrombosis, and the system can be used for quickly removing intra-arterial thrombosis of the lower limbs, so that a good long-term patency rate is obtained after arterial surgery of the lower limbs. Due to its safe, reliable, and efficient clinical advantages, the Straub Rotarex system has been widely used abroad. However, there are few reports on it in China (3, 4). In this study, the therapeutic effects of the Straub thrombectomy system on ALEAE were investigated by comparison with thrombectomy through arteriotomy. Also, the related factors affecting the recurrence of Straub thrombectomy were analyzed, in order to provide a reference for the treatment selection and prognosis of patients with ALEAE.

## Information and Methods

### Research Object

This study was approved by our Ethics Committee and all patients were informed and agreed upon. All the data have been confirmed.

We visited our institutional database to retrospectively screen patients with ALEAE, who were treated in our hospital between April 2018 and April 2021.

#### Inclusion Criteria

All the patients met the AE diagnostic criteria (5) in Guidelines for Diagnosis and Treatment of Lower Limb Arteriosclerosis Obliterans issued by the Chinese Medical Association, including the sudden onset of pain, paresis, paresis, pulseless and pale symptoms in the affected limb of the patient, and the presence of emboli in the affected limb detected by computed tomography (CTA), as well as the existence of organic heart disease, atherosclerosis or a history of arterial embolism. 2) Informed of the study and signed an informed consent form.

#### Exclusion Criteria

Patients with combined distal arterial occlusion; 2) Patients with extensive gangrene of limbs due to excessive embolism time; 3) Those who had a taboo on the thrombolytic drugs used in this study; 4) Patients with moderate to severe anemia; 5) Patients who could not tolerate the surgical protocol selected in this; 6) Patients who cannot be treated with surgery.

### Methods

The patients in observation group were treated with Straub Rotarex system. Preoperative evaluation results of CTA or doppler ultrasound examination of lower limbs were used to select an appropriate surgical approach. During the operation, standard angiography was performed using a sheath tube under DSA. After measuring the diameter of the target lesion vessel, a Rotarex catheter with an appropriate diameter was selected. During the operation, a 4F vertebral arterial catheter was used together with the guidewire of 0.035 inch and 0.018 inch through the occlusion segment, and the GW-0.018 inch guidewire was exchanged, followed by guidewire insertion into a Rotarex catheter and proximal to the occlusion segment. Heparin saline was injected into the catheter lumen before catheter sheath and vascular insertion. Heparin saline was infused into the catheter through the opening in the front of the catheter head. A sterile plastic syringe of appropriate size and without a needle was used to aspirate heparin saline and fill the entire catheter lumen. The catheter tip was moistened with heparin saline to ensure smooth passage through the catheter sheath. Under DSA fluoroscopy, a small forward or backward movement was performed at a speed of 5 mm/s through the occlusive segment and exceeded the occlusive segment by 1 cm. The angiographic evaluation was performed after the whole process was completed. If the residual stenosis is > 30% or there is no straight flow, PTA is feasible or stenting is performed if necessary, and at least one outflow tract of the inferior genicular artery is ensured.

Control group was treated with arterial resection and thrombectomy. Dissection of the distal artery at the site of the embolism was performed, and blocking forceps were used to control blood flow and prevent the thrombus from moving to other sites. The proximal artery was dissected and dissociated, and flow control continued with the forceps. A longitudinal incision was made at the embolization segment. Then, proximal blocking forceps were opened. The embolus was removed with Fogarty catheter and blocked again after the blood flow was cleared. Open the distal blocking forceps. If there is no blood gusher, the distal embolus remains. Suture the vascular wall after unobstructed blood flow at both ends.

Both groups were treated with anti-infection and vasodilation as well as long-term anti-platelet and anticoagulation therapy.

### Observation Index

The curative effects, operation indexes, postoperative complications, and recurrence of patients in the two groups were observed. The criterion for recurrence was the presence of thrombus in the lower extremities on CTA examination.

The efficacy, surgical indicators, postoperative complications, and recurrence of the two groups were observed. Efficacy was evaluated according to Cooley criteria (6). Cure: after treatment, the distal artery pulsation of the affected limb returned to normal, the symptoms of limb ischemia disappeared. Good: after treatment, the pulse of the distal limb was recovered, but weaker than that of the healthy side, and the symptoms of limb ischemia were improved. Poor: after treatment, the distal blood flow of the affected limb did not recover, the limb was compensated by collateral circulation, and there was ischemia Total effective rate= (cured cases + good cases)/total cases × 100%.

### Statistical Methods

The SPSS22.0 software was used for processing. The continuous variable data of experimental data were expressed as mean standard deviation ( ± s) and adopted *t* test. The classified variable data and descriptive analysis were expressed as (%) and adopted χ^2^ test. Multivariate Logistic regression model was used to analyze the significant factors in single-factor analysis. The test level was α = 0.05, and *P*< *0.0*5 was considered statistically significant.

## Results

Finally, according to the inclusion and exclusion criteria, 64 patients were finally included as the research object. There was no significant difference in general information between the two groups (*P* < 0.05), as shown in Table 1.

**Table 1:** Comparison of general data of patients between the two groups.

**Group**	***n***	**Gender (** ***n*** **)**		**Age (years old)**	**Average course of disease (h)**	**Smoking history (** ***n*** **)**		**Basic diseases (** ***n*** **)**				
		**Male**	**Female**			**Yes**	**No**	**Hypertension**	**Diabetes**	**Hyperlipidemia**	**Coronary heart disease**	**Atrial fibrillation**
Control group	32	19	13	66.78 ± 8.94	13.07 ± 3.67	15	17	12	14	9	11	8
Observation group	32	18	14	67.25 ± 8.46	13.25 ± 3.87	13	19	14	15	9	9	7
*χ^2^*/*t*	-	0.064	0.216	0.191	0.254	0.455						
*P*	-	0.800	0.830	0.849	0.614	0.978						

As shown in Table 2, the total effective rate (100%) of the observation group was not significantly different from that of the control group (93.75%) (*P* > 0.05).

**Table 2:** Comparison of curative effect between two groups (*n*, %).

**Group**	***n***	**Cure**	**Good**	**Poor**	**Total response rate**
Control group	32	18 (56.25%)	12 (37.50%)	2 (6.25%)	30 (93.75%)
Observation group	32	21 (65.62%)	11 (34.38%)	0 (0.00%)	32 (100.00%)
χ^2^					1.953
*P*					0.162

As shown in Table 3, the intraoperative blood loss, postoperative off-bed time, and hospital stay time in the observation group were lower than those in the control group, and the operation time and hospitalization expenses were higher than those in the control group. The differences were statistically significant (*P* < 0.05).

**Table 3:** Comparison of surgical indexes between the two groups (*n*, ± s).

**Group**	***n***	**intraoperative blood loss (mL)**	**operative time (min)**	**postoperative off-bed time (d)**	**hospital stay time (d)**	**hospitalization expenses (million Yuan)**
Control group	32	67.73 ± 17.23	95.83 ± 19.45	4.23 ± 1.26	7.19 ± 2.07	5.78 ± 1.07
Observation group	32	56.41 ± 10.14	108.17 ± 22.47	1.54 ± 0.47	3.37 ± 1.03	8.63 ± 1.21
*t*		3.203	2.349	11.315	9.163	9.981
*P*		0.002	0.022	0.000	0.000	0.000

In the observation group, 1 (3.13%) case of reperfusion syndrome occurred after the operation. In the control group, there were 1 (3.13%) case of wound infection, 1 (3.13%) case of hematoma formation, 2 (6.25%) cases of reperfusion syndrome and 2 (6.25%) cases of post-embolization syndrome after operation. The incidence of postoperative complications in the observation group (3.13%) was lower than that in the control group (18.76%) (*P*< *0.0*5). There were 5 cases (15.63%) of postoperative recurrence in the observation group and 6 cases (18.76%) of postoperative recurrence in the control group, and the difference was not statistically significant (*P* > 0.05).

As shown in Table 4, univariate analysis showed that patients with recurrent or non-recurrent embolism had statistically significant differences in hypertension, diabetes, hyperlipidemia, coronary heart disease, and atrial fibrillation (*P* < 0.05).

**Table 4:** Univariate analysis of embolic relapse after treatment with the straub rotarex system (*n*, %).

**Clinical pathological features**		**Recur (*n* = 5)**	**No recur (*n* = 27)**	***χ** ^**2**^*	***P***
Ggender	Male	4 (80.00%)	15 (55.56%)	1.045	0.307
	Female	1 (20.00%)	12 (44.44%)		
Age (year)	≤ 65	2 (40.00%)	17 (62.96%)	0.922	0.337
	>65	3 (60.00%)	10 (37.04%)		
Smoking history	Yes	4 (80.00%)	11 (40.74%)	2.611	0.106
	No	1 (20.00%)	16 (59.26%)		
Hypertension	Yes	4 (80.00%)	8 (29.63%)	4.567	0.033
	No	1 (20.00%)	19 (70.37%)		
Diabetes	Yes	5 (100.00%)	9 (33.33%)	7.619	0.006
	No	0 (0.00%)	18 (66.67%)		
Hyperlipidemia	Yes	4 (80.00%)	5 (18.52%)	7.889	0.005
	No	1 (20.00%)	22 (81.48%)		
Coronary heart disease	Yes	4 (80.00%)	7 (25.93%)	5.468	0.019
	No	1 (20.00%)	20 (74.07%)		
Atrial fibrillation	Yes	5 (100.00%)	3 (11.11%)	4.678	0.031
	No	0 (0.00%)	24 (88.89%)		
Types of postoperative antiplatelet drugs	Aspirin	2 (40%)	14 (51.85%)	0.237	0.626
	Clopidogrel	3 (60%)	13 (48.15%)		
Types of anticoagulants after operation	warfarin	4 (80.00%)	12 (44.44%)	2.133	0.144
	rivaroxaban	1 (20.00%)	15 (55.56%)		

The recurrence of embolism was taken as a dependent variable, and the factors with significant differences in Table 4 were taken as independent variables to be included in the Logistic regression model. The assignments of the dependent variable and independent variable are shown in Table 5.

**Table 5:** Variable assignment table of the risk factors for recurrence of embolism after treatment with the straub rotarex system.

**Variable**	**The assignment**
**Dependent variable**	
Recurrent embolism	No = 0, Yes = 1
**Independent variables**	
Hypertension	No = 0, Yes = 1
Diabetes	No = 0, Yes = 1
Hyperlipidemia	No = 0, Yes = 1
Coronary heart disease	No = 0, Yes = 1
Atrial fibrillation	No = 0, Yes = 1

As shown in Table 6, atrial fibrillation was an independent risk factor for recurrence after treatment with the Straub Rotarex system (*P* < 0.05).

**Table 6:** Multifactor analysis of embolic recurrence after treatment with the straub rotarex system.

**Factors**	**B**	**SE**	**Walds**	**df**	**Sig**.	**EXP (B)**
Hypertension	0.451	0.922	0.531	1	0.077	1.570
Diabetes	0.823	1.291	0.494	1	0.089	2.277
Hyperlipidemia	0.547	1.035	0.511	1	0.084	1.728
Coronary heart disease	0.239	0.482	1.029	1	0.053	1.270
Atrial fibrillation	0.472	0.583	1.388	1	0.025	1.603

## Discussion

The ALEAE is a thrombotic disease caused by cardiac thrombosis, proximal arterial thrombosis and medical implants that fall off and embolize popliteal, femoral, and other lower limb arteries along with blood flow, seriously affecting the lower limb function and quality of life of patients (7, 8). Once embolization is formed, the distal limbs at the embolization site will suffer severe pain due to acute ischemia, which can lead to limb necrosis and gangrene in a short period of time, and even amputation and death and other serious consequences (9, 10). Therefore, timely intervention for patients with ALEAE is the key to improve the prognosis of patients.

### The Straub Rotarex System Is an Effective Treatment for ALEAE After Systemic Therapy

Arteriotomy and thrombectomy are the main methods for clinical treatment of AE, which can effectively improve the ischemic symptoms of patients with AE. However, this method has some practical defects, such as large trauma of thrombectomy, inability to timely intervene in potential artery stenosis, and large reperfusion reaction (11). The Straub Rotarex system is a highly efficient mechanical thrombectomy device consisting of a rotary catheter package, power parts, and control handles. In addition, conventional Seldinger technique puncture and intubation can be used during surgery, with less trauma. Also, due to the guidance of guidewires during the operation, the rotary-cut catheter is easy to reach the position, and it is not easy to damage the blood vessel with the protection of the guide wires. In addition, the catheter and the power component can be separated automatically when the resistance is too high through electromagnetic linkage. If the fibrous tissue is stuck to the catheter head, it can be relieved by reverse rotation, and no lubricant or cooling equipment is required, so the Straub Rotarex system thrombosis cutter is easy to operate and safe to use (12).

In this study, the Straub Rotarex system was compared with thrombectomy in terms of surgical efficacy, surgical indicators, postoperative complications, and embolic recurrence, to explore its clinical application value. The results showed that the therapeutic effect of Straub Rotarex system reached 100%, and the recurrence rate of postoperative embolism was not significantly different from that of open thrombectomy. Also, compared with the thrombectomy and thrombectomy, the operation intraoperative blood loss with the Straub Rotarex system was significantly reduced, the patient's getting out of bed and hospital stay-time were shortened, and the incidence of postoperative complications was reduced. The reason is that the catheter head is shorter, and the knife head does not directly contact the vascular wall, which reduces the stimulation and injury to the vascular wall (13). At the same time, the Straub Rotarex system removes thrombus from the iliac, superficial femoris, popliteal and deep femoris, anterior and posterior tibial arteries, and the beginning of the peroneal artery. The emboli are, then, smashed and transported externally with a built-in blade, effectively preventing distal embolism. Also, the time of intracavitary operation is short, which reduces the risk of intraoperative bleeding (14, 15).

### Atrial Fibrillation Is an Independent Risk Factor for Recurrent Embolic Events After Straub Thrombus Removal System

Patients with ALEAE are at high risk for re-embolization after surgery. In this study, 10 relevant factors including combination of basic diseases, cardiovascular diseases, and drug use were included as variables in the multivariate logistic regression model to analyze the influencing factors of relapse in patients with ALEAE after the Straub Rotarex system treatment, in order to provide a reference for the selection of treatment plan for patients after operation and improvement of patient prognosis. Univariate analysis showed significant differences in hypertension, diabetes, hyperlipidemia, coronary heart disease, and atrial fibrillation between relapsed and non-relapsed patients (*P* < 0.05). Hypertension, diabetes, and hyperlipidemia can all induce the formation of cardiovascular plaques and increase the risk of thrombosis. Coronary heart disease is an incomplete and complete cardiovascular occlusion due to the formation of plaques in the coronary artery. If the plaques are ruptured, thrombus may be formed (16). Atrial fibrillation can lead to blood deposition in the atrium, thus, increasing the risk of thrombosis (17). Cardiac thrombosis is the main embolus source of ALEAE, and rheumatic heart disease and atrial fibrillation are the most common cardiovascular diseases. A cardiac thrombus that falls off and flows with blood in the blood vessels and forms embolism at the arteries of lower limbs will cause the occurrence or recurrence of ALEAE (18). Multivariate analysis showed that atrial fibrillation was an independent risk factor for embolic recurrence after treatment with the Straub Rotarex system. Combined with previous studies, the reason is that atrial fibrillation, as a persistent arrhythmia, can cause irregular heart beating and atrial enlargement, resulting in blood stasis in the atrial and easily forming mural thrombi. Such blood clots are easy to fall off and lead to the recurrence of ALEAE. In addition, patients with atrial fibrillation often present with excessive heart rate, which is likely to lead to the occurrence of unstable heart thrombosis and thus the recurrence of embolism (19, 20).

## Conclusion

In summary, Straub Thrombus removal system is an effective method for the treatment of ALEAE. Although it prolongs the operation time and increases the operation cost as compared with thrombectomy, it effectively improves the operation safety, postoperative life quality, and postoperative recovery, thus, worthy of clinical promotion. Atrial fibrillation is an independent risk factor for recurrent embolism after the Straub Thrombus removal system. Paying attention to the clinical diagnosis and treatment of patients with atrial fibrillation is of great significance for patients to choose a reasonable treatment, prevent recurrence, and improve the prognosis. In addition, the inadequacy of this study is affected by the small sample size, and the feasibility of the results of this study can be further verified by enlarging the sample size in the future.

## Data Availability Statement

The original contributions presented in the study are included in the article/supplementary material, further inquiries can be directed to the corresponding author.

## Ethics Statement

The studies involving human participants were reviewed and approved by the Ethics Committee of our hospital. The patients/participants provided their written informed consent to participate in this study.

## Author Contributions

LZ and QS are the mainly responsible for the writing of the article. HC is mainly responsible for research design. All authors contributed to the article and approved the submitted version.

## Funding

This study was supported by the key research and development program of Shannxi Province (2021SF-322).

## Conflict of Interest

The authors declare that the research was conducted in the absence of any commercial or financial relationships that could be construed as a potential conflict of interest.

## Publisher's Note

All claims expressed in this article are solely those of the authors and do not necessarily represent those of their affiliated organizations, or those of the publisher, the editors and the reviewers. Any product that may be evaluated in this article, or claim that may be made by its manufacturer, is not guaranteed or endorsed by the publisher.
